# Host-like temperature unlocks glucose uptake and metabolism in pathogenic *Leptospira*

**DOI:** 10.1128/mbio.01814-26

**Published:** 2026-10-20

**Authors:** Ryo Ozuru, Michinobu Yoshimura, Deborah A. Powers, Takumi Sonoda, Jason A. Papin, Fumiko Obata, Glynis L. Kolling, Kenji Hiromatsu

**Affiliations:** 1 Department of Microbiology and Immunology, Faculty of Medicine, Fukuoka University 12774, Fukuoka, Japan; 2 Department of Biomedical Engineering, University of Virginia 2358, Charlottesville, Virginia, USA; 3 Division of Bacteriology, Department of Microbiology and Immunology, Faculty of Medicine, Tottori University 13114, Tottori, Japan; Virginia Commonwealth University School of Medicine, Richmond, Virginia, USA

**Keywords:** *Leptospira*, metabolic modeling, glucose metabolism, host-pathogen interactions

## Abstract

Pathogenic *Leptospira* transitions between environmental reservoirs and mammalian hosts, exposing the bacterium to distinct temperature conditions. Although *Leptospira* is generally considered to rely primarily on long-chain fatty acids for growth and to have limited capacity to use glucose, whether host-like temperature alters glucose-associated metabolic capacity remains unclear. Here, we used transcriptome-integrated genome-scale metabolic modeling to examine temperature-dependent metabolic states in pathogenic *Leptospira*. Models contextualized with transcriptomic data from cultures at 37°C predicted increased flux through glucose transport, glucose phosphorylation, and downstream glucose-associated reactions than it did at 29–30°C. Similar temperature-dependent predictions were obtained for another pathogenic *Leptospira* species under *in vitro* conditions. Consistent with these findings, analysis of leptospires cultivated intraperitoneally in dialysis membrane chambers also predicted enhanced glucose uptake and metabolism under host-temperature conditions. *In vitro* validation experiments showed increased bacterial uptake or accumulation of a fluorescent glucose analog, elevated expression of candidate glucose transporter genes, and glucose-dependent enhancement of bacterial growth at 37°C. Treatment with a glucose degradation pathway inhibitor, 2-deoxy-d-glucose, further supported a contribution of glucose-associated processes to proliferation at 37°C. Together, these findings indicate that pathogenic *Leptospira* displays condition-dependent glucose uptake and glucose-associated metabolic activity that become apparent at host-like temperature, as opposed to being glucose-incompetent at environmental-like temperature. This prediction-driven framework refines the conventional view of *Leptospira* carbon metabolism and provides a basis for future studies of glucose-associated metabolic capacity during mammalian infection.

**IMPORTANCE:**

Environmental reservoirs play a central role in the transmission of many bacterial pathogens, yet the metabolic mechanisms that enable adaptation to both environmental and host-associated niches remain largely unknown. Using a prediction-driven framework that combines contextualized genome-scale metabolic modeling with multi-omics analyses and experimental validation, we discovered that pathogenic *Leptospira* activates glucose uptake and metabolism only under host-like conditions. This finding resolves a long-standing misconception arising from studies performed under conventional laboratory conditions and fundamentally revises our understanding of *Leptospira* physiology. More importantly, our study establishes an integrative systems biology strategy for uncovering condition-dependent metabolic traits that would be difficult to identify experimentally alone, with broad applicability to diverse environmentally transmitted pathogens.

## INTRODUCTION

The zoonotic spirochete *Leptospira* can persist outside hosts and survive under various environmental conditions. While *Leptospira* proliferates most efficiently when cultured at 30°C in EMJH or Korthof’s medium (1), pathogenic species also encounter and tolerate the higher body temperature of mammalian hosts, typically around 37°C. Growth at 37°C in these media is accompanied by changes in gene and protein expression (2), suggesting that the physiological and metabolic state of *Leptospira* at host-like temperatures differs from that under standard laboratory culture conditions. Such differences are likely shaped, at least in part, by the availability of carbon sources and by the metabolic capabilities of *Leptospira*. Indeed, numerous studies have demonstrated temperature-dependent changes in *Leptospira* transcript and protein expression (2–9), whereas relatively few have focused specifically on metabolism. Consequently, although growth in EMJH medium is reduced at 37°C relative to 30°C, the metabolic basis of this reduction remains unclear, and it is not known whether it reflects a general decline in metabolic activity or a shift in the pathways that are utilized. Elucidating these temperature-associated metabolic changes may help identify condition-dependent biomarkers and prioritize metabolic processes relevant to severe leptospirosis.

Defining the carbon sources of microorganisms is fundamental to understanding their ecology, host specificity, and physiological responses to environmental and host-related conditions. A key metabolic feature of *Leptospira* is its dependence on long-chain fatty acids as major carbon and energy sources (10, 11). This metabolic feature has been proposed to contribute to *Leptospira* persistence and proliferation in host tissues, including lipid-rich niches (12), although the context-dependent use of alternative carbon sources remains less well understood. Interestingly, while glucose is commonly utilized as a carbon source by many microbes, the zoonotic spirochete *Leptospira* has long been regarded as a notable exception. This bacterium lacks several enzymes involved in the glycolytic pathway, which is the initial route for glucose catabolism (13). Moreover, *Leptospira* exhibits extremely low expression of glucose transporters in EMJH medium (14), resulting in minimal intracellular glucose levels in Korthof’s medium (15). Despite possessing several glycolytic enzymes and a potential for gluconeogenesis, *Leptospira*’s carbohydrate metabolism remains poorly characterized.

Genome-scale metabolic network reconstructions (GENREs) have emerged as powerful tools for investigating microbial metabolism and predicting condition-dependent metabolic capabilities (16). By enabling the prediction of metabolic fluxes and the simulation of systems-level metabolic behavior, GENREs facilitate metabolic engineering and provide mechanistic hypotheses for microbial physiology (16, 17). In the context of infectious diseases, GENREs have been employed to prioritize antimicrobial targets (18) and to explore pathogen metabolism (19, 20). These models have also been used to study pathogen behavior, including the evolution of antibiotic resistance, the production of virulence factors, and host-pathogen interactions (21). For example, GENRE-based analyses of *Pseudomonas aeruginosa* have revealed key metabolic adaptations during chronic lung infections in cystic fibrosis patients, shedding light on disease progression and therapeutic vulnerabilities (22). Although GENREs have previously been constructed for *Leptospira* (23), these models have yet to be fully leveraged to examine its temperature-dependent metabolic responses under host-like conditions.

**Fig 1:**
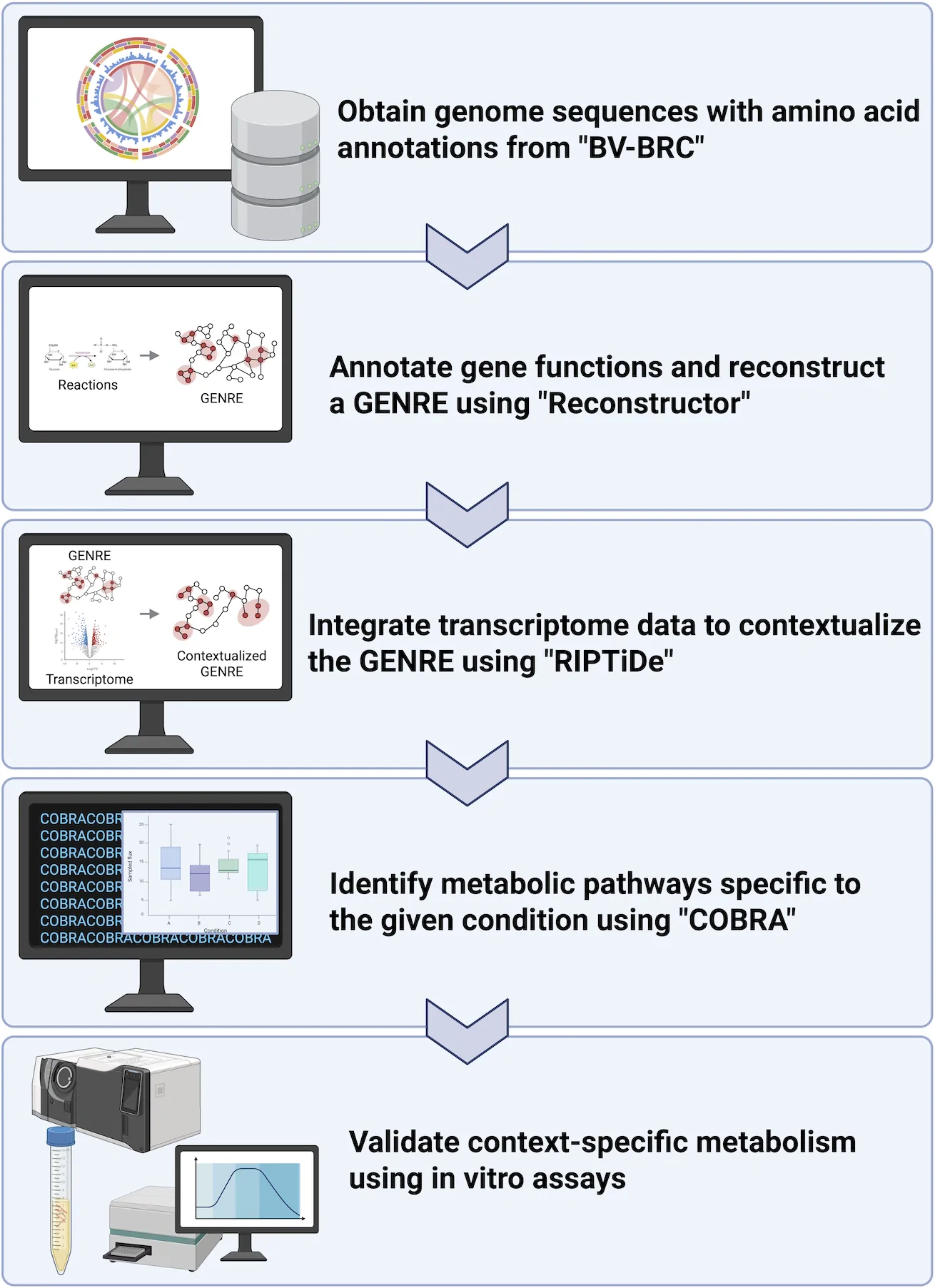
Workflow for genome-scale metabolic network reconstruction, context-specific analysis, and validation. Created in BioRender (R. Ozuru, 2026, https://BioRender.com/k7boo1a).

In this study, we constructed GENREs of pathogenic *Leptospira* to investigate how its metabolic network responds to changes in external temperature. We integrated publicly available transcriptomic data sets derived from cultures grown at different temperatures and from host-like conditions into the models. This approach enabled us to contextualize the *Leptospira* metabolic model and to generate condition-specific predictions that better reflect transcriptomic states under physiologically relevant conditions. Using this framework, we aimed to identify model-predicted metabolic changes associated with temperature shifts and host-associated environments. Furthermore, we experimentally examined the relationship between these predicted metabolic changes, particularly those related to glucose-associated metabolism, and physiological phenotypes in *Leptospira*, including glucose uptake and growth dynamics (Fig. 1). Through this integrative approach, we sought to evaluate whether GENREs can uncover condition-dependent metabolic capacities in pathogenic *Leptospira*.

## RESULTS

### Temperature-dependent metabolic changes predicted by transcriptome-integrated GENREs of pathogenic *Leptospira*

**Fig 2:**
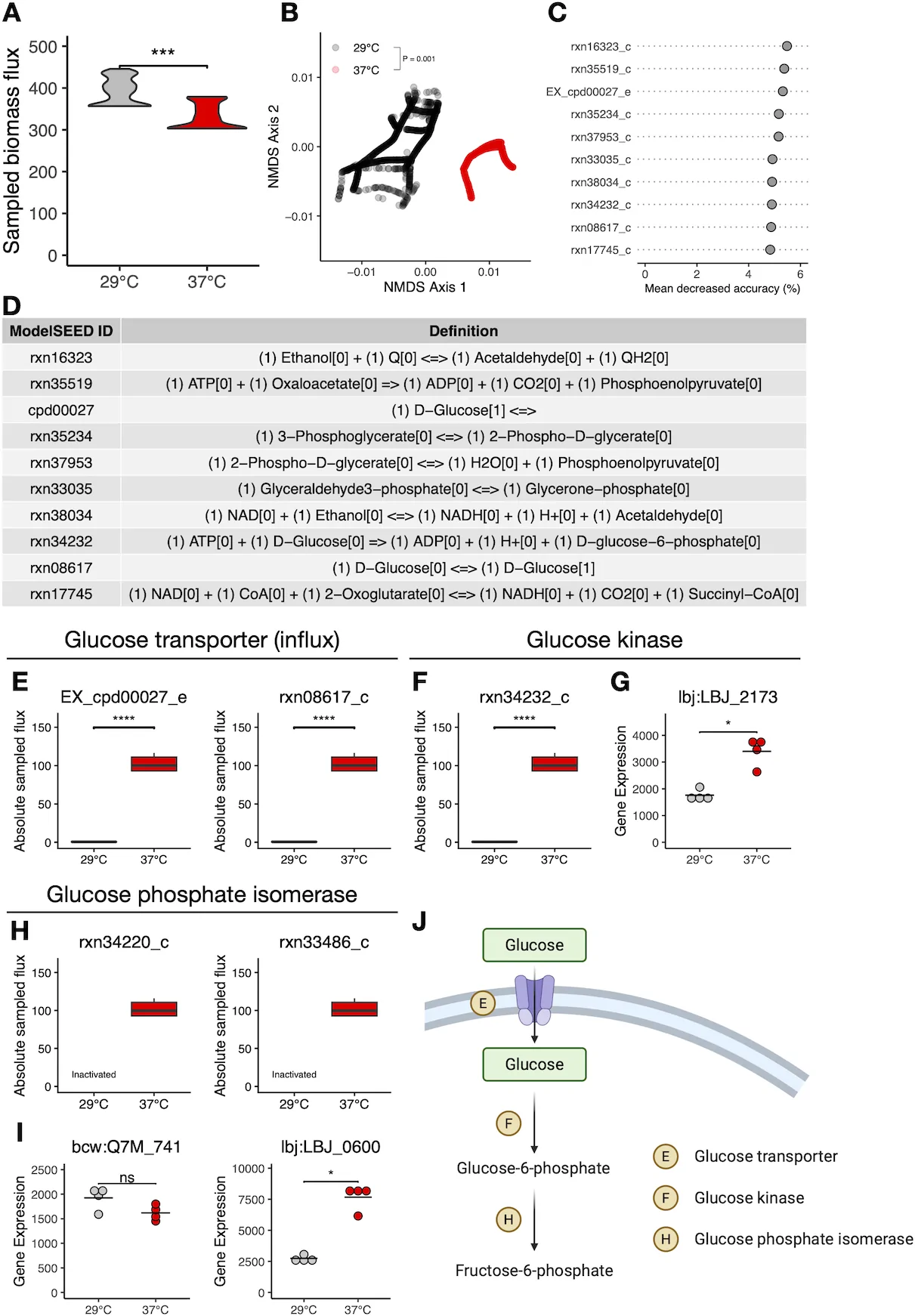
Comparison of metabolic characteristics of contextualized GENREs under different temperature conditions. (**A**) Biomass levels at each temperature condition. (**B**) Comparison of reaction flux distributions for metabolic reactions shared across all temperature conditions. (**C**) List of metabolic reactions in reaction IDs that distinguish temperature conditions was identified using a machine learning approach. Reaction IDs are from the ModelSEED database. All the actual flux plots (29°C vs 37°C) are shown in Fig. S1. (**D**) Reaction IDs and definitions listed in panel **C**. (*n*) indicates the number of molecules involved in the reaction. Compartments [0] and [1] represent the intracellular and extracellular spaces, respectively. (**E and F**) Flux balance analysis results for selected reactions at different temperatures from panel **C** and Fig. S1. Absolute values of sampled fluxes are shown. Each plot title corresponds to a reaction ID from the ModelSEED database. (**G**) Expression of the gene associated with rxn34232_c. (**H**) Flux balance analysis results for selected reactions that were inactive at 29°C but became active at higher temperatures. (**I**) Expressions of the genes associated with rxn34220_c and rxn33486_c. (**J**) Overview of glucose metabolism in *Leptospira*. Panels **E**, **F**, and **H** correspond to specific reactions indicated in this pathway. Created in BioRender (R. Ozuru, 2026, https://BioRender.com/lwavfld). Statistical significance was evaluated using the Wilcoxon rank-sum test (**A, G, and I**), the Dunn test with Bonferroni correction (**E and F**), and permutational ANOVA (permANOVA) (**B**). *P < 0.05, ***P < 0.001, and ****P < 0.0001. ns: not signiticant.

Draft GENREs of pathogenic *Leptospira* were constructed using Reconstructor (24), a tool that automatically builds draft metabolic models based on the amino acid–annotated FASTA files. The accession numbers of the genome data used, along with genome size, gene count, and model characteristics—such as the number of genes, reactions, and metabolites, as well as MEMOTE (25) scores—are summarized in Table S1. The MEMOTE score is a comprehensive quality metric that assesses the structural completeness, annotation quality, consistency, and simulation readiness of genome-scale metabolic models. To refine these models and generate condition-specific metabolic predictions, we integrated publicly available transcriptomic data from the NCBI Gene Expression Omnibus (GEO). Specifically, we used data set GSE168507 (26), which contains transcriptomic profiles of the highly pathogenic *L. borgpetersenii* strain JB197 cultured in HAN medium at 29°C and 37°C. Data integration was performed using RIPTiDe (27), an algorithm that incorporates transcriptomic data to inform reaction activity and flux distributions within GENREs. Flux balance analysis (FBA) of the context-specific models predicted reduced growth potential at 37°C compared to 29°C (Fig. 2A), which is consistent with the known optimal growth temperature of *Leptospira* in EMJH medium around 30°C. Dimensionality reduction of the predicted reaction flux profiles separated the two temperature conditions (Fig. 2B). To identify reactions that contributed most to this temperature-associated separation, we applied a random forest classifier, which highlighted key metabolic reactions distinguishing the two context-specific models (Fig. 2C and D; Fig. S1).

### Model-predicted activation of glucose-associated reactions under temperature upshift in *Leptospira*

Because *Leptospira* exhibits a reduced predicted flux through the biomass objective reaction at 37°C (Fig. 2A), it is expected that the magnitude of metabolic fluxes also decreases under this condition. Nevertheless, only three reactions (glucose transporter; EX_cpd00027_e and rxn08617_c, and glucose kinase; rxn34232_c) showed an increase in absolute flux values at 37°C, and we therefore focused on these reactions (Fig. 2E and F). The glucose kinase reaction (rxn34232_c) corresponds to the gene ID lbj:LBJ_2173 in the model, which also exhibited increased expression at 37°C according to the original RNA-seq data (Fig. 2G). In addition, we examined reactions that were removed at 29°C but retained at 37°C during RIPTiDe contextualization of both models (Fig. 2H). These reactions were associated with glucose phosphate isomerase, which converts glucose 6-phosphate to fructose 6-phosphate (Fig. 2J, reaction H), with two steps, mediated by rxn34220 and rxn33486, corresponding to the conversion to beta-glucose 6-phosphate and then to fructose 6-phosphate, respectively (Fig. 2H). Both reactions are associated with bcw:Q7M_741 or lbj:LBJ_0600 genes in the model, but only lbj:LBJ_0600 showed increased expression at 37°C in the original RNA-seq data (Fig. 2I). Together, these findings indicate that the transcriptome-integrated model predicts a 37°C-associated increase in glucose uptake, phosphorylation, and downstream conversion capacity toward fructose-6-phosphate in pathogenic *Leptospira* (Fig. 2J).

### Predicted glucose-associated metabolic changes in additional *Leptospira* species *in vitro* and under host-mimicking conditions

To assess whether the glucose-associated response observed in JB197 was conserved across other *Leptospira* strains and species, we first examined the corresponding genes in *L. borgpetersenii* serovar Hardjo strain HB203, which was reported in the same transcriptomic study as JB197. Unlike JB197, HB203 did not show a consistent increase in expression of these genes at 37°C (Fig. S2). We then conducted transcriptome-integrated metabolic analyses for two additional *Leptospira* species using publicly available data sets and their corresponding metabolic models. First, we integrated transcriptomic data from *L. interrogans* serovar Manilae strain L495 cultured in EMJH medium at 30°C and 37°C (GSE92976 [28]) into its metabolic model and compared the predicted flux distributions (Fig. S3). This analysis suggested a temperature-associated pattern similar to that predicted for JB197. Next, we analyzed transcriptomic data from a dialysis membrane chamber (DMC) model (GSE53818 [29]), in which *L. interrogans* serovar Copenhageni was encapsulated in a dialysis membrane and implanted into the rat peritoneal cavity to mimic *in vivo* infection. A similar glucose-associated pattern was also predicted under these host-like conditions (Fig. S4, DMC vs *in vitro* [IV]). These modeling results suggest that similar glucose-associated metabolic changes can occur in multiple pathogenic *Leptospira* species under host-like conditions, although the transcriptional response was not uniform across all strains examined.

### Temperature-dependent glucose uptake and glucose-supported growth by *Leptospira* at 37°C

**Fig 3:**
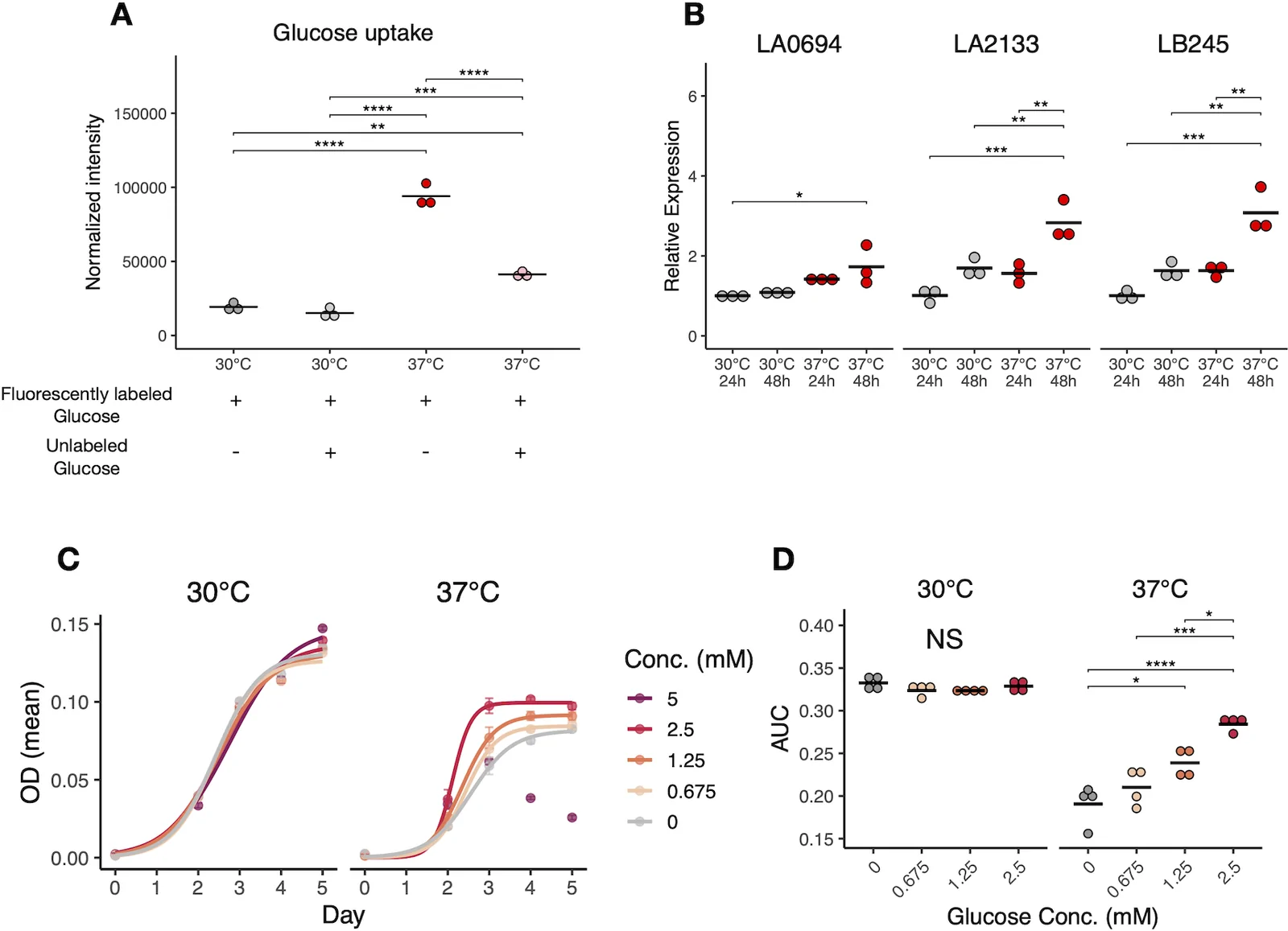
Comparison of glucose uptake and metabolic capacity of *Leptospira* at different temperatures. (**A**) Glucose uptake capacity at various temperatures, normalized by bacterial cell number. Competition assays were performed by co-incubating fluorescently labeled glucose with unlabeled glucose. (**B**) Expression levels of known glucose transporter genes at 30°C vs 37°C. (**C**) Growth curves of *Leptospira* in EMJH medium supplemented with glucose. (**D**) The area under the curve (AUC) from panel **C**. Statistical significance was evaluated using one-way ANOVA followed by Tukey’s HSD test. *P < 0.05, **P < 0.01, ***P < 0.001, and ****P < 0.0001.

**Fig 4:**
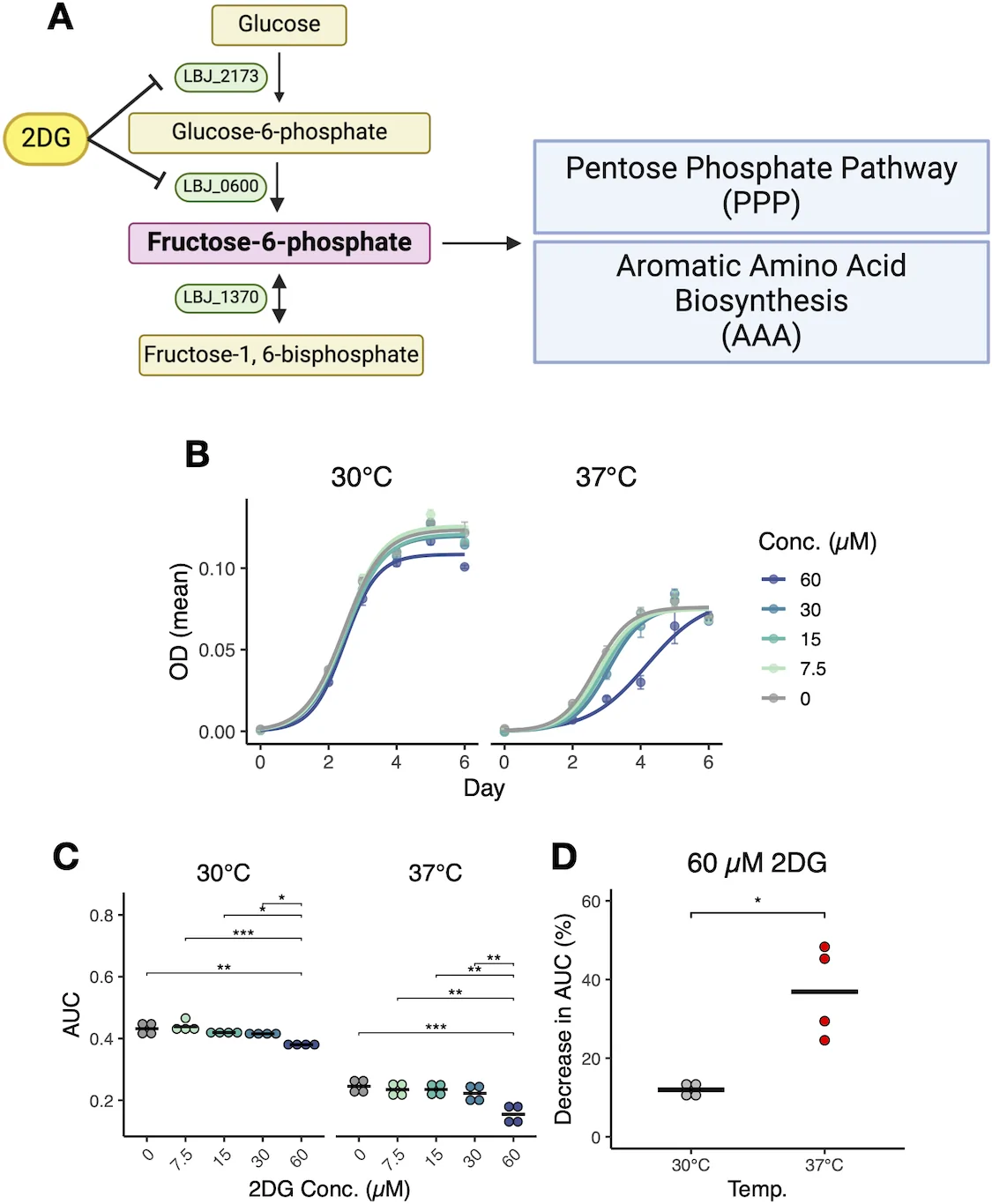
Inhibition of *Leptospira* growth at different temperatures by targeting glycolytic and gluconeogenic pathways. (**A**) Schematic diagram of glycolytic pathways active at 37°C. Fructose 6-phosphate, which is intensively synthesized under these conditions, is expected to serve as a precursor for the pentose phosphate pathway (PPP) and aromatic amino acid (AAA) biosynthesis. Enzymes of *L. borgpetersenii* strain JB197 catalyzing each reaction are shown in green (LBJ_XXXX). Created in BioRender (R. Ozuru, 2026, https://BioRender.com/rg4lq23).(**B**) Growth curves of *Leptospira* at different temperatures in EMJH medium supplemented with 2-deoxyglucose (2DG). (**C**) The area under the curve (AUC) from panel **B**. (**D**) Decrease in the AUC. The AUC values without 2DG at each temperature were normalized to 100%. Statistical significance was evaluated using one-way ANOVA followed by Tukey’s HSD test (**C**) and Wilcoxon’s rank-sum test (**D**). *P < 0.05, **P < 0.01, and ***P < 0.001.

*Leptospira* is generally believed to rely primarily on long-chain fatty acids as carbon sources, with limited capacity to uptake and metabolize glucose. To re-evaluate this assumption, we investigated glucose uptake at different incubation temperatures using a fluorescence-labeled glucose analog (Fig. 3A). Notably, *Leptospira* exhibited significantly greater uptake of fluorescent glucose at 37°C compared to 30°C. This uptake was competitively inhibited by the addition of 10% unlabeled glucose, supporting the specificity of the transporter-mediated process. Based on the hypothesis that increased glucose uptake would be accompanied by upregulation of the corresponding transporter genes, we quantified the expression of previously reported glucose transporter genes (14) by RT-qPCR (Fig. 3B). At 48 h, the expression of LA2133 and LB245 was significantly elevated at 37°C compared with that at 30°C. To assess whether glucose availability affects bacterial proliferation in a temperature-dependent manner, we supplemented EMJH medium with glucose and compared growth curves at 30°C and 37°C (Fig. 4C and D). At 30°C, glucose supplementation had no significant effect on proliferation. In contrast, at 37°C, proliferation increased with glucose concentrations up to 2.5 mM. These results physiologically support the model prediction that host-like temperature is associated with an increase in glucose uptake capacity and glucose-supported growth in *Leptospira*.

### Contribution of glucose-associated metabolic activity to *Leptospira* proliferation under host-like temperature

Fructose 6-phosphate, a model-predicted downstream product of glucose-associated reactions, serves as a precursor for the pentose phosphate pathway and the aromatic amino acid biosynthesis pathway (Fig. 4A). These pathways are key metabolic branches that provide reducing power, nucleotides, and precursors for protein and coenzyme synthesis, all of which support bacterial growth. We therefore hypothesized that perturbation of this metabolic node would affect *Leptospira* proliferation, particularly under conditions in which glucose-associated reactions are predicted to be more active. To test this hypothesis, we performed *in vitro* growth assays in the presence of a pathway inhibitor: 2-deoxy-d-glucose (2DG), a glucose analog that can interfere with glycolysis (Fig. 4A). Although growth inhibition by 2DG at 60 µM was also observed at 30°C (Fig. 4B and C), the reduction in proliferation was more pronounced at 37°C (Fig. 4D). These findings support the idea that glucose-associated metabolic activity contributes to pathogenic *Leptospira* proliferation under host-like temperature conditions. Together, these data are consistent with a temperature-dependent contribution of glucose-associated metabolic activity to pathogenic *Leptospira* proliferation *in vitro*.

## DISCUSSION

**Fig 5:**
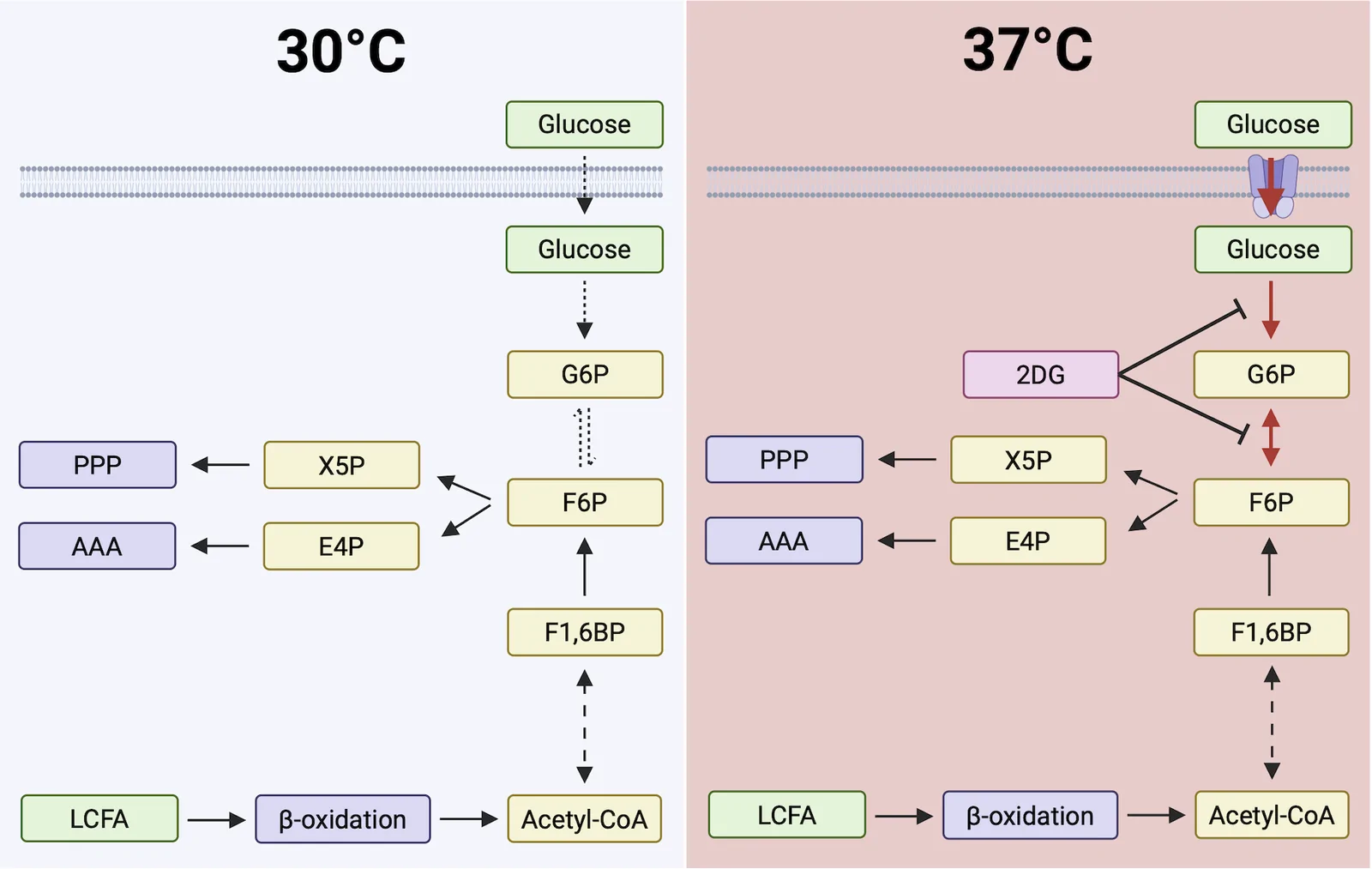
Graphical summary of this study. Left, at 30°C, the optimal temperature for *Leptospira* proliferation, glucose uptake and metabolism are inactive. Instead, pyruvate is synthesized from alternative carbon sources such as glycerol for energy production. Right, at 37°C, glucose uptake and its conversion to fructose 6-phosphate (F6P) are enhanced. The resulting F6P is channeled into the pentose phosphate pathway (PPP) and aromatic amino acid (AAA) biosynthesis to produce bacterial components. Inhibition of glycolytic pathways by 2DG impairs *Leptospira* growth. Created in BioRender (R. Ozuru, 2026, https://BioRender.com/qp9tha8).

*Leptospira* has long been considered to rely primarily on long-chain fatty acids, with limited capacity to use glucose as a carbon source. In this study, we provide evidence that pathogenic *Leptospira* displays temperature-dependent glucose uptake, glucose-associated metabolic activity, and glucose-supported growth when cultured at elevated temperatures (Fig. 5). This temperature-dependent glucose-associated phenotype may contribute to growth under host-like conditions (Fig. S4). Most previous investigations assessing *Leptospira* metabolism, including those focused on carbon source utilization, were conducted at 30°C, the optimal *in vitro* growth temperature in EMJH/Korthof’s medium. Our findings highlight the importance of considering host body temperature when interpreting *Leptospira*’s metabolic behavior.

Previous genome analysis of *L. interrogans* serovar Copenhageni indicated that this organism possesses nearly all the enzymes comprising the glycolytic pathway (30). The authors concluded that the limited utilization of glucose was more likely attributable to constraints in glucose transport than to deficiencies in the downstream metabolic pathway. This interpretation is consistent with our finding that glucose transporter-encoding genes LA2133 and LB245, referred to as *putP*-like genes (14) and distinct from LA0694, exhibited increased expression at host-like temperature (Fig. 3B). Increased expression of these genes may contribute to enhanced glucose uptake (Fig. 3A). Although *putP* is generally annotated as a proline-sodium symporter, homologs in another bacterium have been shown to transport glucose (31). It is therefore possible that the *putP*-like genes in *Leptospira* contribute to condition-dependent glucose uptake, although their substrate specificity and transport activity remain to be experimentally validated.

The question of whether *Leptospira* utilizes glucose as a carbon source has been debated since the organism’s initial discovery. In the late 1910s, Noguchi reported that the addition of glucose to the culture medium did not affect the growth of *Leptospira* (32), leading to the long-standing view that the bacterium has limited capacity to utilize glucose. In the 1960s, Ellinghausen demonstrated that glucose supplementation significantly stimulated the growth of a *Leptospira* isolate obtained from frog kidney tissue, particularly when combined with polysorbate 80 in the culture medium (33). However, the isolate used in that study originated from an amphibian host (*Rana pipiens*), and Ellinghausen observed that growth was optimal at 29°C and severely impaired at 34°C. In our study, we found that candidate glucose transporter gene expression, glucose uptake, and glucose-associated metabolic activity increased in a mammalian-pathogenic *Leptospira* at 37°C. Notably, these measurements were made in glucose-free EMJH medium, indicating that this response does not require the prior presence of glucose and is instead modulated by temperature itself.

The conditions under which fastidious strains are propagated provide further support. HAN medium (34), formulated to enable isolation directly from animal hosts at 37°C, contains approximately 3.5 mM glucose from its DMEM/Ham’s F-12 component, whereas EMJH is glucose-free and supports optimal growth at approximately 30°C. This suggests a possible basis for its performance: organisms recovered from a mammalian host and maintained at 37°C would be positioned to exploit a carbon source present *in vivo* but absent from conventional formulations. Since HAN differs from EMJH in other respects, this correspondence is supportive rather than conclusive. Together, these observations shift the question from whether *Leptospira* can use glucose to the physiological conditions under which glucose-associated metabolic capacity becomes apparent.

Several studies have examined aspects of glucose-associated physiology in *Leptospira*. Yanagihara et al. characterized glucose 6-phosphate isomerase, which is one of the few experimentally validated components of the glycolytic pathway in this organism (35). Additional evidence for glucose responsiveness comes from studies of chemotaxis (36, 37). Although chemotaxis is not directly linked to glucose transport or catabolism, the existence of a system capable of sensing or responding to glucose gradients is consistent with the possibility that glucose detection and acquisition have physiological relevance in *Leptospira*. Together with these earlier observations, our findings suggest that glucose-associated traits in *Leptospira* may be conditional rather than absent, becoming more apparent under specific physiological parameters such as host-like temperature.

Relatively little attention has been given to how temperature affects carbon source utilization in *Leptospira*. In contrast, numerous studies have examined temperature-dependent changes in *Leptospira* transcription and translation, motivated in part by the discrepancy between its optimal *in vitro* growth temperature and mammalian host body temperatures. Shifts from environmental temperatures (20–30°C) to host-associated conditions (37–39°C) induce substantial transcriptional reprogramming, affecting cell wall and membrane biogenesis, chemotaxis, motility, and virulence-associated genes, including temperature-responsive regulation of Lig proteins (3, 4). These observations raise the possibility that *Leptospira* exhibits distinct metabolic activities under environmental and host-like conditions. In the current study, we identified model-predicted metabolic changes under host-like temperature and *in vivo*–proximal condition and experimentally supported their association with glucose uptake and glucose-supported growth *in vitro* (Fig. 2 and 3; Fig. S4). Further investigation of temperature-dependent metabolic phenotypes during mammalian infection may inform biomarker discovery and metabolic target prioritization for leptospirosis.

Public transcriptomic data sets involving temperature shifts (26, 28) and DMC conditions (29) were used to contextualize the *Leptospira* metabolic model. The L495 temperature model (Fig. S3) exhibited less pronounced changes than the JB197 temperature model and Fiocruz L1-130 DMC model (Fig. 2; Fig. S4). This difference may partly reflect experimental conditions. The L495 data set was obtained 18 h after the temperature shift in glucose-free EMJH medium, whereas the other data sets captured responses over several days, and the JB197 data set was generated in glucose-containing HAN medium. Although our uptake measurements in glucose-free EMJH medium indicate that glucose uptake capacity is induced by temperature rather than substrate exposure, glucose availability during growth may influence its transcriptional manifestation. These models remain limited by the use of public transcriptomic data not specifically designed for this question and by the assumption of metabolic steady state inherent to constraint-based modeling. Nevertheless, integration across multiple conditions revealed a recurrent pattern of temperature- and host-like condition-associated glucose-related predictions. Importantly, these predictions resulted from integrating transcriptional data with network topology, stoichiometry, and mass-balance constraints rather than from differential expression of individual genes alone. Their subsequent experimental support by glucose uptake and inhibition assays suggests that the modeling approach provided mechanistic insights beyond transcriptomic comparison alone.

*Leptospira* synthesize erythrose 4-phosphate from fructose 6-phosphate, which serves as a precursor for the biosynthesis of aromatic amino acids (13), and these amino acids contribute to membrane protein stability and virulence-associated functions (38–42). Thus, predicted fructose 6-phosphate (F6P)-linked metabolic changes may have physiological consequences beyond growth. We propose two non-mutually exclusive hypotheses linking glucose metabolism to host adaptation. First, increased flux through the pentose phosphate pathway, fueled by glucose catabolism, may enhance NADPH supply and thereby support resistance to oxidative stress encountered within the host. Second, glucose-derived carbon entering downstream pathways, including F6P-linked aromatic amino acid biosynthesis, may increase the supply of precursors required for survival and proliferation in the host environment. Under this view, glucose utilization would not directly activate specific virulence factors, but would instead support the metabolic fitness and physiological adaptability underlying virulence. These hypotheses could be tested experimentally by inhibiting glucose transport or glucokinase activity and assessing the resulting effects on oxidative stress resistance, expression of virulence-associated genes, and survival within the host.

This study has several limitations. First, because our analyses focused primarily on pathogenic *Leptospira* strains associated with mammalian hosts, it remains unclear whether the temperature-dependent glucose uptake and metabolism are restricted to pathogenic strains or are more broadly conserved across the genus. Some nonpathogenic species reportedly grow preferentially at 37°C on glucose-containing HAN agar rather than at 29°C (43), suggesting that this phenotype may not be restricted to the pathogenic clades. However, because HAN differs from conventional media in components other than glucose, the contribution of glucose remains unclear. Comparative studies including nonpathogenic strains will define how temperature-dependent carbon utilization varies across *Leptospira* species. Second, not all available transcriptomic data sets could be incorporated. We analyzed *L. borgpetersenii* serovar Hardjo strain HB203, reported alongside JB197 (26), but RIPTiDe contextualization produced no model capable of sustaining biomass flux at either temperature, precluding flux sampling. Although the underlying reconstruction could produce all biomass precursors before contextualization, pruning based on HB203 expression data rendered several basal metabolites unproducible. Thus, transcriptome-integrated modeling depends on the expression profile, and apparently comparable data sets may not be equally amenable to contextualization. The use of a fixed biomass objective across conditions is another limitation, as temperature-dependent changes in cell morphology are not represented; indeed, some *L. biflexa* grown in HAN medium showed similar growth rates at 29°C and 37°C despite increased cell length at 37°C (44). Third, this study did not directly trace the intracellular fate of glucose-derived carbon. Fluorescent glucose analogs support temperature-dependent glucose uptake or accumulation but do not define the pathways utilized by imported glucose. Similarly, 2DG sensitivity is consistent with a contribution of glucose-associated processes to proliferation at 37°C, but it does not establish the essentiality of specific reactions. Genetic or biochemical validation is also required to determine whether LA2133 and LB245 function as glucose transporters. Finally, because much of this study is based on *in vitro* experiments and transcriptome-integrated metabolic modeling, whether this phenotype operates during mammalian infection remains unresolved.

In conclusion, this study supports a model in which pathogenic *Leptospira* displays condition-dependent glucose uptake and glucose-associated metabolic activity at host-like temperature, challenging the long-standing view of limited glucose utilization. By combining transcriptome-integrated metabolic modeling with *in vitro* validation, we identified a temperature-dependent metabolic phenotype that refines the conventional view of *Leptospira* carbon metabolism. Future studies using stable isotope tracing, comparative analyses across the genus, animal models, and clinical specimens will be required to define the relevance in biochemical, evolutionary, and *in vivo* aspects of the glucose-associated metabolic capacity.

## MATERIALS AND METHODS

### Bacterial strain, culture conditions, and reagents

*Leptospira interrogans* serovar Manilae strain L495 was used in this study. L495 was cultured in EMJH medium (BD Biosciences, Franklin Lakes, NJ, USA) at either 30°C or 37°C under static conditions. Bacterial counts were determined using a Petroff-Hausser counting chamber (Sunlead Glass Corp., Saitama, Japan) under a dark-field microscope (BX-53, Olympus, Tokyo, Japan). Other reagents used in this study were as follows: 2-Deoxy-d-glucose (Cat# 10722-11, Nacalai Tesque Inc., Kyoto, Japan).

### Genome-scale metabolic network reconstruction in pathogenic *Leptospira*

Genome-scale metabolic models of pathogenic *Leptospira* were constructed using Reconstructor version 1.0.76 (24). Amino acid sequence data annotated with PATRIC (45) used as input and obtained from the Bacterial and Viral Bioinformatics Resource Center (BV-BRC 3.47.8) (46). During model reconstruction, both EMJH medium and the complete medium required by Reconstructor were specified (Table S2). All reaction identifiers in the resulting models were derived from the ModelSEED database (47).

### Integration of public transcriptome data to contextualize GENREs

Reconstructed GENREs were integrated and contextualized using publicly available transcriptomic data with the RIPTiDe algorithm (27). Briefly, RIPTiDe maps gene expression values onto individual metabolic reactions through the gene–protein–reaction (GPR) associations encoded in each model and assigns a weight to each reaction based on its corresponding transcript abundance. Using these weights, RIPTiDe identifies a parsimonious flux distribution that is maximally consistent with the transcriptional state while maintaining biomass production within a specified range of its optimal value. This procedure was applied separately to each condition to generate context-specific GENREs, and the feasible solution space of each resulting model was sampled by running simulations with random sampling; the resulting flux distributions were compared and analyzed. Reaction bounds were used at their default values as implemented in Reconstructor and COBRApy. During RIPTiDe contextualization, the minimum objective (biomass) flux fraction was set to RIPTiDe’s default value of 0.8. The feasible solution space of each resulting model was then sampled 1,000 times, exceeding RIPTiDe’s default sample size of 500 to improve the robustness of the flux distributions obtained. All linear optimization problems were solved using Gurobi (Gurobi Optimization, LLC). The following RNA-seq data sets were used: (i) GSE168507 (26), *L. borgpetersenii* serovar Hardjo strain JB197 incubated in HAN medium at 29°C and 37°C; (ii) GSE92976 (28), *L. interrogans* serovar Manilae strain L495 cultured in EMJH medium at 30°C and 37°C; and (iii) GSE53818 (29), *L. interrogans* serovar Copenhageni cultured in a dialysis membrane chamber (DMC) model embedded in the rat peritoneal cavity or *in vitro* in EMJH medium at 30°C. All RNA-seq data used in this study were obtained from previously published data sets and were not reprocessed from raw sequencing files. Important reactions were selected based on random forest feature importance, expressed as the mean decrease in accuracy, as described in a previous study (48). A larger mean decrease in accuracy indicates that permutation of the corresponding reaction has a greater negative effect on classification performance.

### Fluorescent-labeled glucose uptake assay

Glucose uptake by *Leptospira* was quantified using the Glucose Uptake Assay Kit-Green (UP02, Dojindo, Kumamoto, Japan), following the manufacturer’s instructions. L495 strain was incubated for 48 hours in EMJH medium at 30°C or 37°C. The cells from 1 mL of culture were collected by centrifugation at 2,800 × *g* for 15 min at 4°C and washed twice with 500 µL of the washing buffer supplied with the kit. The washed cells were then incubated with the fluorescent glucose analog for 15 min at 30°C or 37°C, after which unincorporated label was removed by two further washes with 500 µL of washing buffer (13,000 × *g*, 2 min, 4°C). The cells were finally resuspended in 500 µL of washing buffer for measurement. To confirm the specificity of uptake, parallel incubations were performed in the presence of 10% unlabeled glucose. Fluorescence-conjugated glucose uptake was measured using a SPARK 10M microplate reader (TECAN, Männedorf, Switzerland) in fluorescence detection mode (excitation, 470 nm; emission, 520 nm). Fluorescence intensity was normalized to bacterial cell number and expressed as relative uptake.

### Growth curve assay and fitting

Cultures were inoculated at 10^7^ leptospires/mL and incubated at 30°C or 37°C. Bacterial growth was monitored using an iMark microplate reader (Bio-Rad, Hercules, CA, USA) by measuring absorbance at 450 nm at 24-h intervals. For glucose supplementation experiments, EMJH medium was supplemented with glucose. For growth inhibition assays, 2-deoxy-d-glucose (2DG) was added to EMJH. Throughout the experiments, cultures were also examined by dark-field microscopy (BX-53; Olympus) to confirm typical cell morphology, active motility, and the absence of visible contamination. Growth curves were generated based on absorbance data using the growthcurver package in R (version 0.3.1 [49]). Based on a logistic growth model, the area under the curve (AUC) was estimated.

To relate absorbance to cell number, calibration curves were constructed at each temperature. Cultures were serially diluted and enumerated by dark-field microscopy using a Thoma cell counting chamber (Sunlead Glass Corp.n), and cell density was plotted against absorbance at 450 nm (Fig. S5). An absorbance of 0.1 corresponded to approximately 7.4 × 10^8^ leptospires/mL at 30°C and 7.7 × 10^8^ leptospires/mL at 37°C.

### Gene expression analysis

*L. interrogans* strain L495 was cultured in EMJH medium at 30°C and 37°C for 24 and 48 h. The cells were harvested by centrifugation, and total RNA was extracted using the Direct-zol RNA Microprep kit (R2063, Zymo Research Corp., Irvine, CA, USA). cDNA was synthesized using the ReverTra Ace qPCR RT Master Mix with gDNA Remover (FSQ-301, TOYOBO Co., Ltd., Osaka, Japan). Quantitative real-time PCR was performed using the CFX Connect Real-Time PCR Detection System (Bio-Rad) with Thermo Scientific SYBR Green qPCR Master Mix (A66732, Thermo Fisher Scientific Inc., Waltham, MA, USA). Gene expression levels were quantified using the ΔΔCt method, with 16S rRNA as the internal reference gene. Primers used in this study are listed in Table S3.

### Statistical analysis

All statistical analyses in this study, including the Welch’s *t*-test, one-way ANOVA followed by Tukey’s HSD test, Wilcoxon rank-sum test, Dunn’s test with Bonferroni correction, and permutational ANOVA (PERMANOVA), were performed using R (version 4.4.0). Normality was assessed using the Shapiro–Wilk test or Q–Q plots prior to applying parametric tests. Statistical significance was defined as *P*  <  0.05.

## Data Availability

All code used in this study, the generated genome-scale metabolic models, and the raw and processed data supporting the findings are available in a GitHub repository (https://github.com/O2U/Lepto-Gluc-Metab).

## SUPPLEMENTAL MATERIAL

Supplemental material mbio.01814-26-s0001.docx Fig. S1 to S5, Table S1, and Table S2 and S3 captions.

Table S2 mbio.01814-26-s0002.xlsx Medium compounds used in this study.

Table S3 mbio.01814-26-s0003.xlsx Primer list.

ASM does not own the copyrights to Supplemental Material that may be linked to, or accessed through, an article. The authors have granted ASM a non-exclusive, world-wide license to publish the Supplemental Material files. Please contact the corresponding author directly for reuse.
