# Supplementary material for: Host-like temperature unlocks glucose uptake and metabolism in pathogenic Leptospira
Source: mBio. 2026 Oct 20;17(11):e01814-26. doi: 10.1128/mbio.01814-26 (PMC13753783; doi:10.1128/mbio.01814-26)
Supplement: Supplementary file 1 [file mbio.01814-26-s0001.docx]

**Fig. S1 Flux balance analysis results for all reactions selected in Fig. 2C.** Each plot title corresponds to a reaction ID from the ModelSEED database. All boxplots representing 29 °C data are shown in white, while those for 37 °C data are shown in red. Raw sampled flux values are shown without transformation. Exchange reactions EX_cpd00027_e is defined with efflux from the cell as the positive direction; negative values therefore indicate uptake, and a shift toward more negative values indicates increased uptake. Note that Fig. 2E shows the absolute value of the sampled flux for the same reactions.

**Fig. S2. Expression of glucose-associated genes in *L. borgpetersenii* serovar Hardjo strain HB203 at 29 °C and 37 °C.** Expression levels of genes corresponding to glucose-associated reactions identified in the JB197 model were compared between cultures grown at 29 °C and 37 °C. Individual points represent biological replicates (n = 4). Statistical significance was evaluated using Welch’s t-test. *P < 0.05, ***p* < 0.01, ****p* < 0.001; ns, not significant.

**Fig. S3 Flux balance analysis results for glycolytic and gluconeogenic reactions at different temperatures in *Leptospira interrogans* serover Manilae strain L495.** Each plot title corresponds to a reaction ID from the ModelSEED database. Statistical significance was evaluated using the Wilcoxon rank-sum test. *P < 0.05, ***p* < 0.01, ****p* < 0.001, and *****p* < 0.0001

**Fig. S4 Flux balance analysis results for glycolytic and gluconeogenic reactions under different culture condition in *Leptospira interrogans* serovar Copenhageni strain Fiocruz L1-130.** Each plot title corresponds to a reaction ID from the ModelSEED database. IV denotes *in vitro* condition, whereas DMC mimics infected host condition. Statistical significance was evaluated using the Wilcoxon rank-sum test. *P < 0.05, ***p* < 0.01, ****p* < 0.001, and *****p* < 0.0001

**Fig. S5 Relationship between absorbance and cell density of *Leptospira* cultures at 30 °C and 37 °C.** Cultures grown at 30 °C (blue) or 37 °C (red) were serially diluted, and cell density was determined by dark-field microscopy using a Thoma cell counting chamber and plotted against absorbance at 450 nm. Each point represents [the mean of n independent counts / a single dilution]; lines indicate linear regression (30 °C, R² = 0.999; 37 °C, R² = 0.997). An absorbance of 0.1 corresponded to approximately 7.4 × 10⁸ leptospires/mL at 30 °C and 7.7 × 10⁸ leptospires/mL at 37 °C.

**Table S1 | Genome data and the model characteristics in this study.**

| Strain | GenBank Accession# | Genome size Mbp | Genome genes | Model genes | Model metabolites | Model reactions | MEMOTE score |
| --- | --- | --- | --- | --- | --- | --- | --- |
| JB197 | CP000350  CP000351 | 3.876 | 4,197 | 952 | 1,266 | 1,113 | 84% |
| Fiocruz L1-130 | AE016823  AE016824 | 4.627 | 4,550 | 994 | 1,290 | 1,151 | 84% |
| L495 | OEJX00000000 | 4.615 | 4700 | 1,007 | 1,294 | 1,156 | 84% |

**Table S2: Medium compounds used in this study (separate XLSX file)**

**Table S3: Primer list used in this study (separate XLSX file)**
